# Role of Natural Phenolics in Hepatoprotection: A Mechanistic Review and Analysis of Regulatory Network of Associated Genes

**DOI:** 10.3389/fphar.2019.00509

**Published:** 2019-05-24

**Authors:** Priyanka Saha, Anupam Das Talukdar, Rajat Nath, Satyajit D. Sarker, Lutfun Nahar, Jagajjit Sahu, Manabendra Dutta Choudhury

**Affiliations:** ^1^Department of Life Science & Bioinformatics, Assam University, Silchar, India; ^2^Centre for Natural Products Discovery, School of Pharmacy and Biomolecular Sciences, Liverpool John Moores University, Liverpool, United Kingdom; ^3^Department of Mycology and Plant Pathology, Institute of Agricultural Sciences, Banaras Hindu University, Varanasi, India

**Keywords:** gene network, hepatoprotection, phenolics, liver disease, *in silico* analysis, mechanism, natural product

## Abstract

The liver is not only involved in metabolism and detoxification, but also participate in innate immune function and thus exposed to frequent target Thus, they are the frequent target of physical injury. Interestingly, liver has the unique ability to regenerate and completely recoup from most acute, non-iterative situation. However, multiple conditions, including viral hepatitis, non-alcoholic fatty liver disease, long term alcohol abuse and chronic use of medications can cause persistent injury in which regenerative capacity eventually becomes dysfunctional resulting in hepatic scaring and cirrhosis. Despite the recent therapeutic advances and significant development of modern medicine, hepatic diseases remain a health problem worldwide. Thus, the search for the new therapeutic agents to treat liver disease is still in demand. Many synthetic drugs have been demonstrated to be strong radical scavengers, but they are also carcinogenic and cause liver damage. Present day various hepatic problems are encountered with number of synthetic and plant based drugs. Nexavar (sorafenib) is a chemotherapeutic medication used to treat advanced renal cell carcinoma associated with several side effects. There are a few effective varieties of herbal preparation like Liv-52, silymarin and Stronger neomin phages (SNMC) against hepatic complications. Plants are the huge repository of bioactive secondary metabolites viz; phenol, flavonoid, alkaloid etc. In this review we will try to present exclusive study on phenolics with its mode of action mitigating liver associated complications. And also its future prospects as new drug lead.

## Introduction

The liver is labeled as the biggest glandular organ that controls diverse physiological and chemical processes in human body. In other words, it plays a central role in metabolic control and detoxification involving metabolism of lipids, carbohydrates, alcohol, and a wide range of drugs as well as toxins (Aseervatham et al., 2018). The liver also participates in innate immune function (Gao et al., 2008). Interestingly, the liver has the unique ability to regenerate and completely recoup from most acute, non-iterative situations (Mosedale and Watkins, 2017; Oliva-Vilarnau et al., 2018). However, multiple conditions, e.g., hepatitis, chronic alcohol consumption, frequent use of antibiotics associated medications, and also even non- alcoholic fatty liver disease, can affect the regenerative efficacy of the hepatocytes which become totally dysfunctional (Forbes and Newsome, 2016), generally witnessed by visible hepatic scaring, apoptosis and entering into the most severe cirrhosis. The liver, when witness to such atrocities, ultimately loses its vitality and thus imbalances the normal metabolic phenomenon, leading to many other fatal conditions (Branco et al., 2016; Defronzo et al., 2016). Despite considerable amounts of research which have been carried out aiming at cure various hepatic ailments across the world, limitations still exist in finding more effective hepatoprotective drugs than the currently available medications. Moreover, fewer medications promise restoration effects.

The Mediterranean-style diet which covers the immense geographical area adjoining the Mediterranean Sea focuses on the use of root legumes, vegetable, fruits, nuts and seeds predominantly (Tuck and Hayball, 2002). Presently there is an arising concern or interest, rather, n exploring the positive effects of a plant-based diet for mitigating various chronic diseases including several hepatic ailments like hepatic cirrhosis, hepatic ulcerative syndrome and fibrosis. It is noteworthy that the Mediterranean diet, which has been allied with many health benefits, is characterized by a high intake of fruits, vegetables and nuts containing several bioactive natural products of plants. One such dietary component common in plant-based diets are natural phenolics, which are particularly plentiful not only in fruits, whole grains, vegetables, and legumes but equally in coffee, tea, cocoa, and also in red wine.

Phenolics are a bulky and heterogeneous group of phytochemicals containing phenol rings, and are divided into several groups, viz; phenolic acids, flavonoids and lignin. Fruits such as pears, grapes, apples, and a range of berries naturally contain good amounts of polyphenols (250–400 mg in 100 g). The most frequent phenolic acids are ferulic acid and caffeic acid of which the major phenolic compounds in coffee and cereals, respectively, are comprised. The most studied stilbene is resveratrol, found in red wine and grape products (Veberic et al., 2008). Other main dietary sources of natural phenolics comprise of chocolate, green tea, and whole grains. Polyphenol contains abundant antioxidants in the diet and these act as natural scavengers for toxic elements and, thus, their intake has been directly connected with a reduced frequency of several hepatic ailments, particularly hepatocellular carcinoma in humans (Turati et al., 2014). Phenolics also exhibit anti-inflammatory effects and influences hepatotoxicity through altered mechanisms discussed in detail in the subsequent paragraphs.

Thus, herbal approach, an alternative to the conventional protocol with a touch of a therapeutic essence, remains a valid option. These strategies, in most cases, not only target the disease but also contain minimal side effects. The majority of the available synthetic drugs for liver diseases are found to be strong pro-oxidant scavengers, but their long-term uses may cause inflammation (Rani et al., 2016; González-Ponce et al., 2018) and cancer. A noteworthy instance is the use of tiopronin, which increases the risk of liver injury ten-fold with its long-term treatment (Tang et al., 2014; Wan and Jiang, 2018). Another well-illustrated detrimental combination is ribavirin and interferon-α (IFN-α), a common medication in liver-related diseases, which is seen to affect hepatitis C patients. Taking into consideration such complications and the high cost of available medicines, researchers are inclined to utilize natural product-based alternative medications for liver diseases, which will have better efficacy, cost-effectiveness, and lower or no toxicity (Zhang et al., 2013; Seeff et al., 2015).

**Figure 1:**
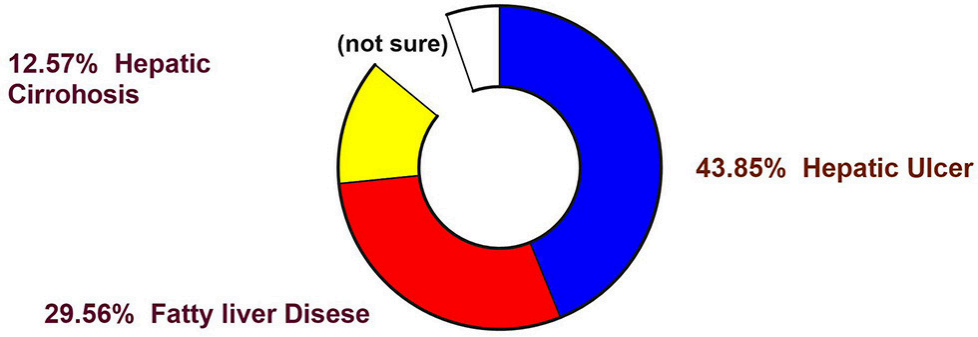
Statistical representation of mortality (in percentage) from various diseases in human (Finkelstein et al., 2012).

**Figure 2:**
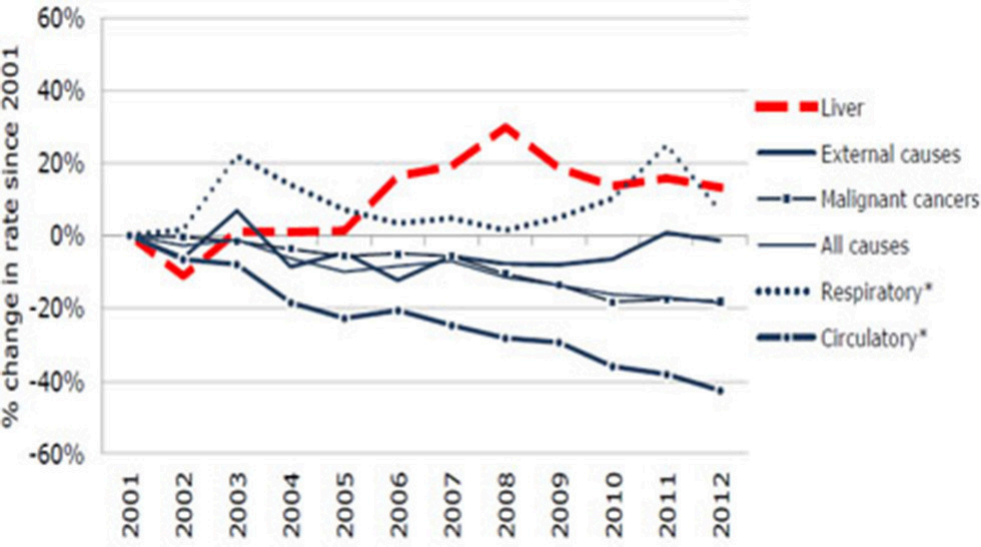
Occurrence and prevalence of various liver diseases worldwide.

It is evident from the reports of the WHO (WHO 2016) that non-communicable diseases were the cause of 68% of all global death in 2012 (Figure 1), rising from 60% in 2000. Hepatic complications have turned out to be multifactorial diseases that affected a population of around almost 600 million in 2014 (Figure 2), and it is likely to amplify by about 33% over the next two decades (Finkelstein et al., 2012; Dhilleswara Rao et al., 2017).

Hepatic ailment results in anomalous hypertrophy, expressed phenotypically with surplus adiposity, body fatness and brawny genetic correlation, while its constituent to basal metabolic index and associated health hazard of obesity have also been reported (Locke et al., 2015; Stender et al., 2017).

Various hepatic problems are encountered with a number of synthetic as well as plant-based drugs. Nexavar is a chemotherapeutic drug generally prescribed for complex renal carcinoma (Ravaud et al., 2016; Decker et al., 2017). It is additionally used to treat liver carcinoma. Known adverse effects of Nexavar include dry skin, itching, skin rash, nausea, vomiting, diarrhea, patchy hair loss, loss of appetite, stomach pain, dry mouth, hoarseness, and tiredness (Schmidinger and Bellmunt, 2010). Sorafenib is the first multi-kinase inhibitor (TKI) approved for the treatment of advanced hepatocellular cancer (HCC) metastatic renal cell cancer (RCC), and well-differentiated radioiodine-resistant thyroid cancer (DTC) (Monsuez et al., 2010). It demonstrates targeted activity on several families of receptor and non-receptor tyrosine kinases that are involved in angiogenesis, tumor growth and metastatic progression of cancer (Adnane et al., 2006). Sorafenib is a well-known antihepatotoxic drug available in market but the product of its metabolism has been seen to be toxic, affecting other parts of the body with long-term exposure resulting in renal and pancreatic failure (Randrup Hansen et al., 2017; Balderramo et al., 2018).

**Figure 3:**
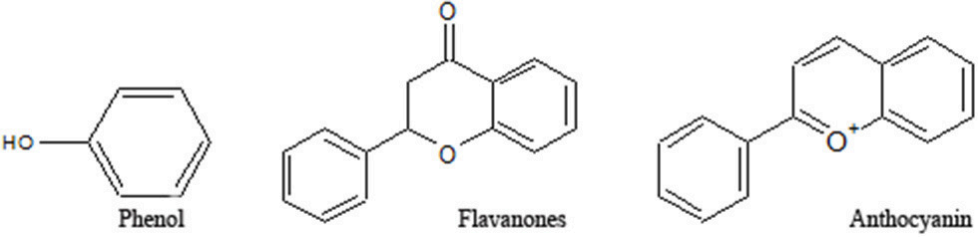
Structures of various groups of phenolic compounds (Hussain, 2016; Mandal et al., 2017; Xi et al., 2018).

A few efficient varieties of herbal preparation like Liv-52, silymarin (Kolasani et al., 2017) and Stronger neomycin phages (SNMC) are in attendance against hepatic complications. All the candidates come up with notable complications. Silymarin is not found effective against chronic liver disease as it fails to modulate the metabolic condition of the liver along with cellular recovery. An effective Japanese preparation like SNMC (Ghiliyal and Bhatt, 2017) also fails to improve the clinical status of liver cirrhosis despite its prominent anti-inflammatory and cytoprotective efficacy. It is successfully used against hepatocellular carcinoma (Luo et al., 2015). Liv-52 is used quite effectively against hepatic damages (Stickel and Hellerbrand, 2015). However, it also fails to demonstrate clinical efficacy in alcoholic liver damages. Various research involving techniques with increasing efficacy of the phytochemicals like nanotechnology, proteomics, and transcriptomics are evident, and efforts being made with herbal preparations are somewhat successful too (Patil et al., 2018). Following the lead of these interesting results, further attempts should be initiated to overcome all the odds of existing drugs, and an initiative may proceed with plant-based natural products. The plants are an enormous repository of bioactive secondary metabolites viz; alkaloids, flavonoids, phenol, etc (Figure 3). This review presents an account of studies on phenolics, with an emphasis on its mechanisms toward hepatotoxicity. Emphasis have been given to understand various pathways through which phenolics exihibit their efficacy. Furthermore, a gene networking model has been constructed in order to gain a clear concise idea of the ways in which natural phenolics contribute to mitigating various hepatic ailments.

## Methods

With an aim to evaluate the actual sceneries of phenolic compounds for the treatment of various hepatic diseases, a search on the metabolic disease Library and PubMed has been performed matching the keywords “hepatic disease inhibitors treatment,” “target therapy,” and “Hepatic carcinoma,” limited to the English written literature, but with no restriction of time. It was examined and the titles of 202 relevant papers were retrieved.

While performing the search of abstracts and full-text research papers, all unrelated and less important ones were discarded. A selection of the most recent, well-illustrated, full-text, and cited articles were considered regarding similar types of research work from the same institutes at different points in time. We have tried not counting the research papers, whose abstract or full-text is not obtainable. The references for significant and relevant papers have been further sought for other pertinent articles. After such an illustrative survey, around 160 latest bioactivity reports of phenolic compounds mitigating hepatic diseases were brought into light, and around 38 clinical trials have been retrieved gratifying the indispensable criteria for analysis.

The gene networking model and connectivity model were developed by analyzing all the available reports on the hepatoprotective activity of the natural phenolics using the online software Cirrcon and Cytoscape version 3.6.1.

## Natural Molecules as Potent Antihepatotoxic Agent

Plant secondary metabolites are well-known for their efficacy in the treatment, as well as prevention, of various fatal diseases. Plant phenolics, e.g., coumarins, flavonoids, lignans, stilbenoids, and tannins, have been studied extensively to provide scientific rationale behind their potential usage against various human ailments. Phenolics are the target group for this review article and subsequent discussions will revolve around exploring the chemical nature and modes of action of these compounds (Smith, 2017; Stander et al., 2017).

**Figure 4:**
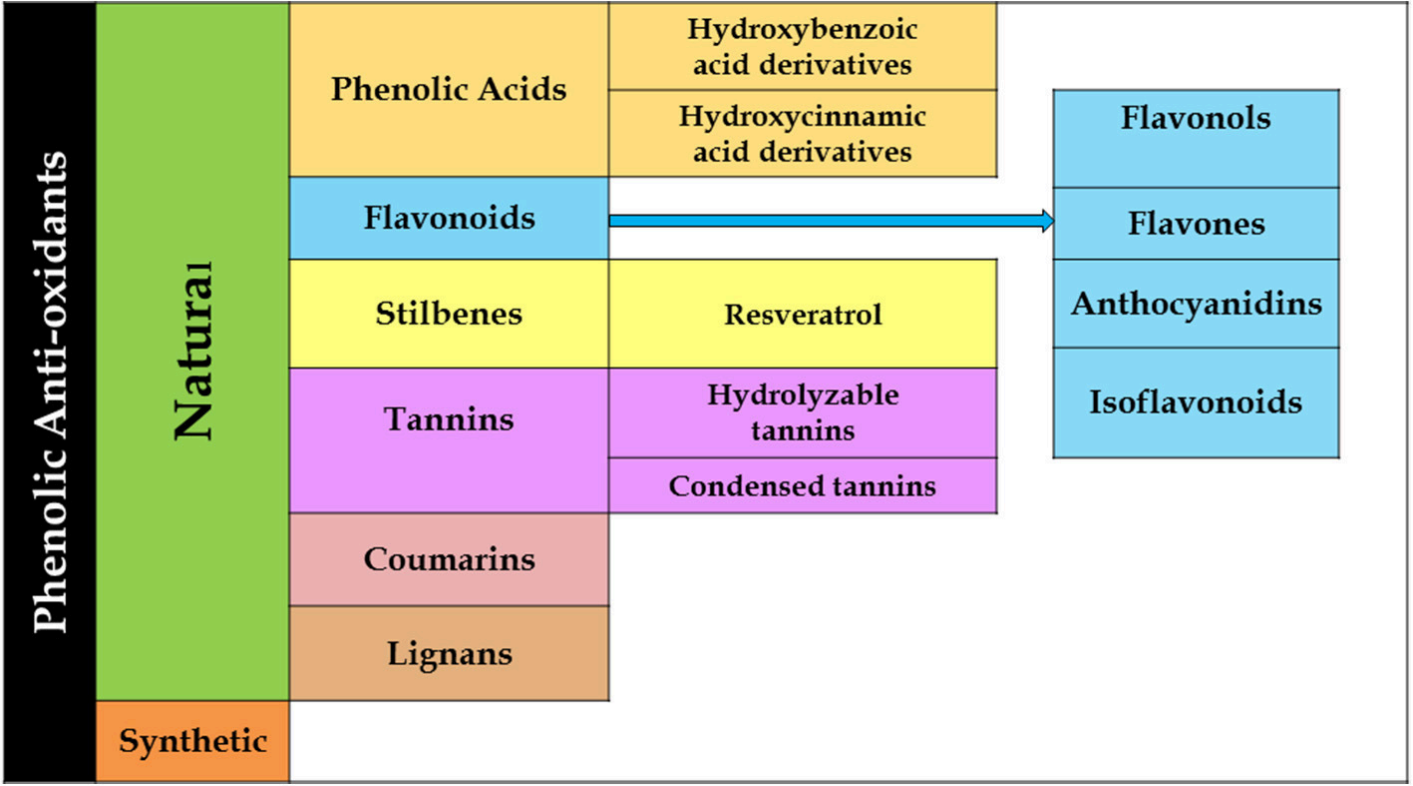
Flowchart showing various descendants of the phenolic groups.

Phenolics constitute a major portion of all secondary plant metabolites discovered to date, and there are about 8,000 of such compounds, in both conjugated and free-form, which are distributed in all parts of the plant. Phenolics are generally biosynthesized from acetyl CoA, shikimate, and amino acids (Cseke et al., 2016; Saltveit, 2017). Plant phenolics include simple phenols, phenolic acids, coumarins, lignans, flavonoids, diaryl-alkanoids, stilbenoids, proanthocyanins, tannins, and anthocyanins some alkaloids (Figure 4).

Natural products have been an integral part of medicine since ancient times where around 400 different species of plant and animal origins were then listed. According to the WHO, plant-based therapies are regularly used in places where minerals, plants are common and easily available. Such therapies are used by around 88% of the world's population, who depend on natural products for their primary health care regime. Though the term “drug discovery” sounds contemporary, the story and origin of drug discovery dates back many centuries. Thus, present day uses of plants for both “lead molecule” discovery, which confirms their activity as active natural molecules, and for their structural analog, proves them to be an ideal drug candidate.

Natural products drug discovery is a hot topic as of recent years, with a comeback in mainstream drug discovery protocols. Such comeback is welcomed by academics and pharma companies, mainly due to inherent chemical diversities in natural products, and ease in identification and separation techniques. Noteworthy among natural products are alkaloids, carbohydrates, glycosides, and terpenoids. Phenolics are most studied due to their antioxidant activities. The phenolic moiety is responsible for various pharmacological effects (Sarker and Nahar, 2007).

Phenolic acids are mainly represented as derivatives of benzoic acid and cinnamic acid. The methyl ester of the phenol ring imparts a pharmacophore, which is responsible for interacting with various protein targets present in cell membranes. Gallic, ellagic, vallic, procatecutic, procoumaric, and caffeic acids are important representatives of hydroxyl benzoic acid and hydroxyl cinnamic acid, which are the product of the condensation reaction of phenols under sunlight (Ahmad et al., 2016; De Beer et al., 2017). Flavonoids on the other hand, biosynthesised from cinnamic acids, have two benzene rings (ring A and ring B), and an apyrrole ring (ring C). Plant flavonoids are generally classified into flavan, flavanone, flavanol, flavone, and flavonols (Sarker and Nahar, 2007). Often there are prenylations, glycosidations and conjugation with other ring systems or natural skeletons, as well as dimerisations and oligomerisations which diversify flavonoid structures and provide new pharmacophores. Quercetin, hesperidin, diosmetic, myrectin, and kaempferol are just a few notable examples imparting biological properties (Hussain, 2016; Brodowska, 2017).

**Figure 5:**
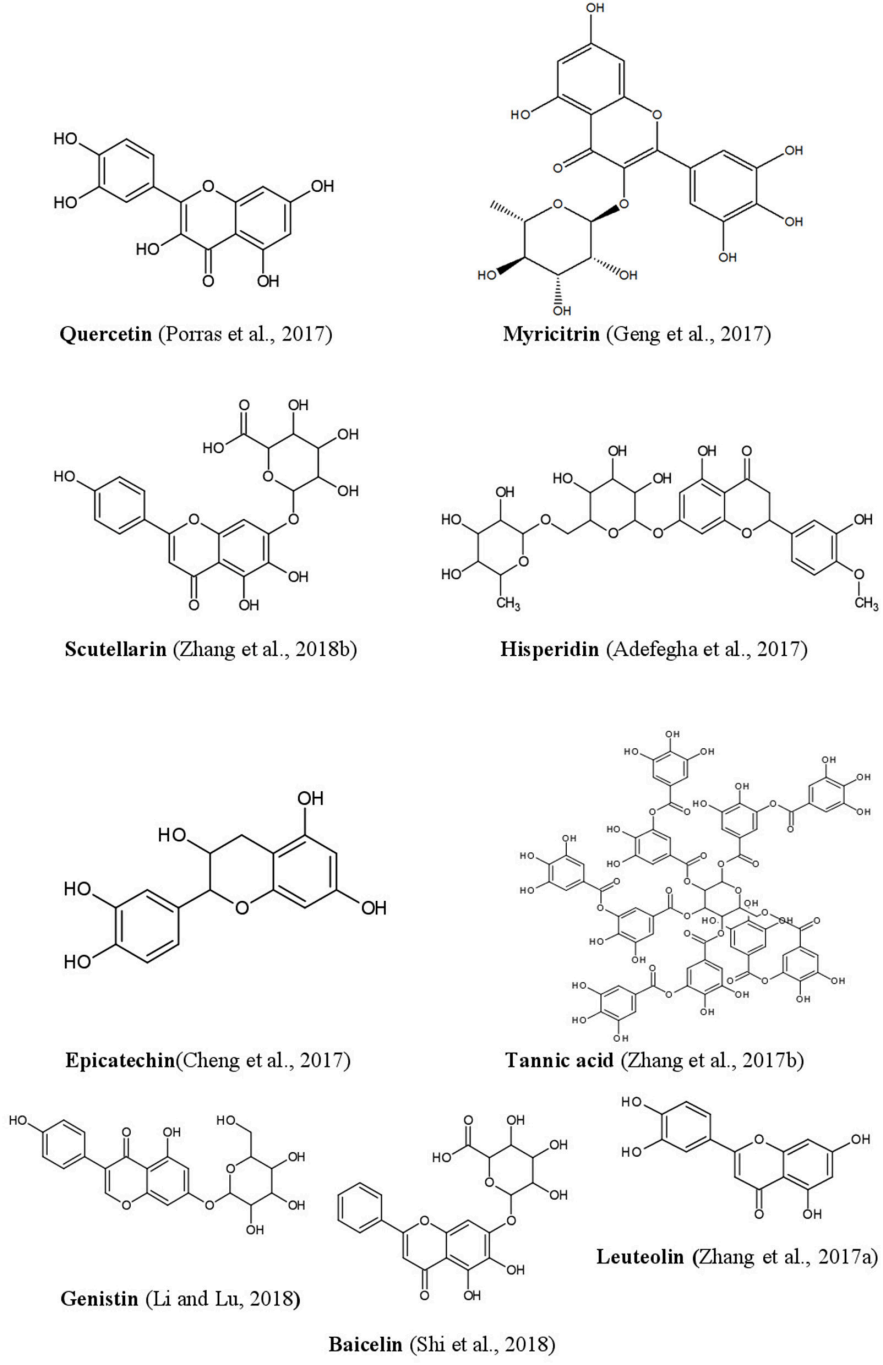
Structures of some bioactive phenolics acting as hepatoprotective compounds.

**Figure 6:**
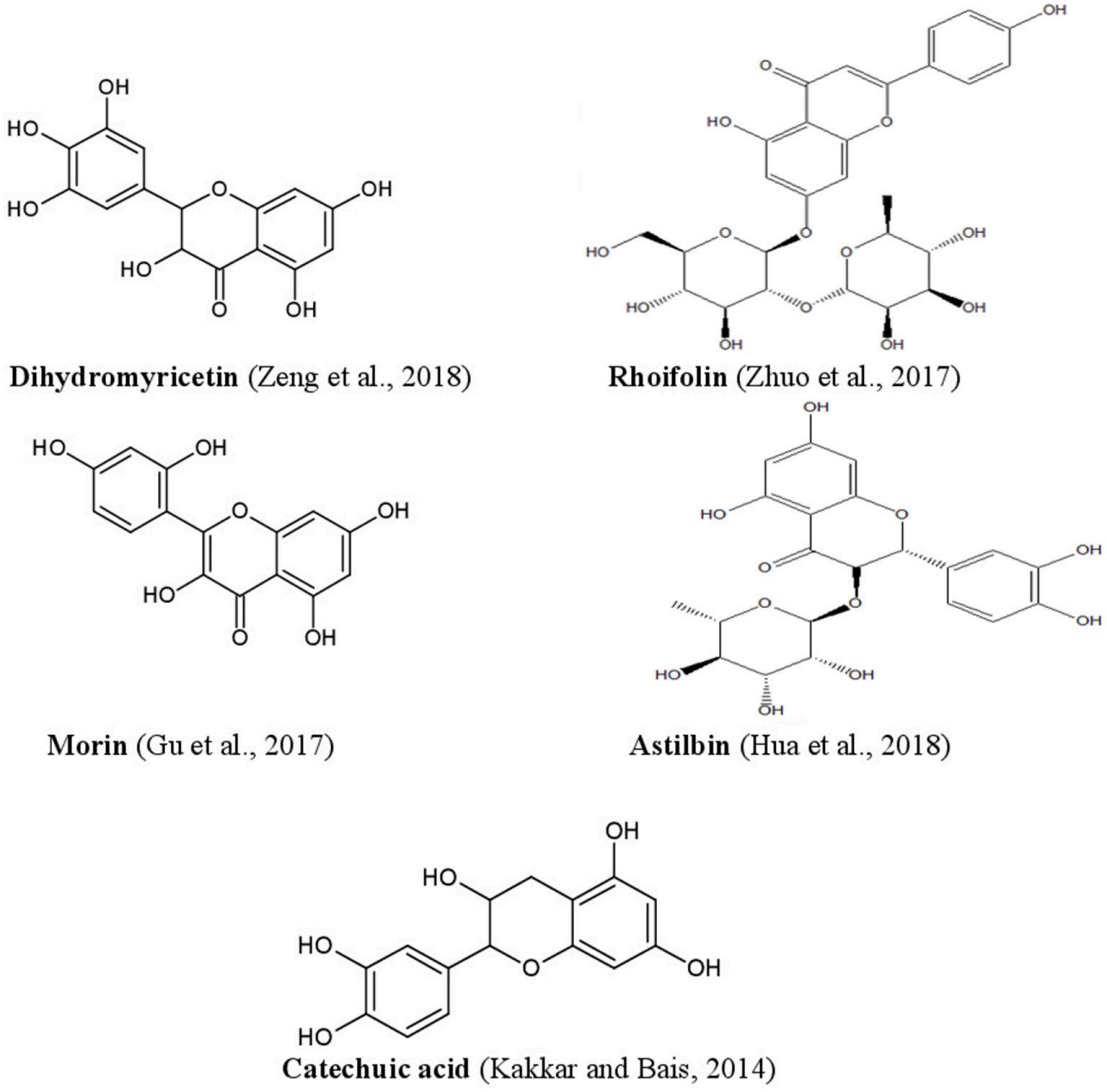
Structures of some bioactive phenolics acting as hepatoprotective compounds.

Anthocyanidins and anthocyanins are normally plant pigments. Anthocyanidins are grouped into 3-hydroxyanthocyanidins, 3-deoxyanthocyanidins, and *O*-methylated anthocyanidins. On the other hand, anthocyanins are in the forms of anthocyanidin glycosides and acylated anthocyanins (Sarker and Nahar, 2007). The most common types of anthocyanidins are cyanidin, delphinidin, pelargonidin, peonidin, petunidin, and malvidin (Figures 5, 6) (Wallace and Giusti, 2015; Chorfa et al., 2016; Mäkilä et al., 2016; Stein-Chisholm et al., 2017). The site of glycosylation in anthocyanidins is usually at C-3 (Kay et al., 2017; Rodriguez-Amaya, 2018; Zhang et al., 2018). Acylated anthocyanins are presented with *p*-coumaric acid, ferulic acid and caffeic acid with attached sugar molecules, in addition to simple acetyl groups (Sigurdson et al., 2017; Zhao et al., 2017).

## Phenols are Important as Prospective Drug Leads

Phenolic compounds are known for their diverse chemical structures, common antioxidant and specific anti-inflammatory actions. They offer protection against oxidative damages by donating hydrogen or electron to free radicals and thus, in this process, they aid in stabilizing cell membrane networks and inhibiting the formation and expression of inflammatory cytokines like tumor necrosis factor alpha (TNF-α), Transforming Growth Factor beta (TGF-β) and varieties of interleukins (IL-6, IL-2, IL-8) (Parhiz et al., 2015; Taofiq et al., 2015; Zhang and Tsao, 2016; Zhen et al., 2016).

**Figure 7:**
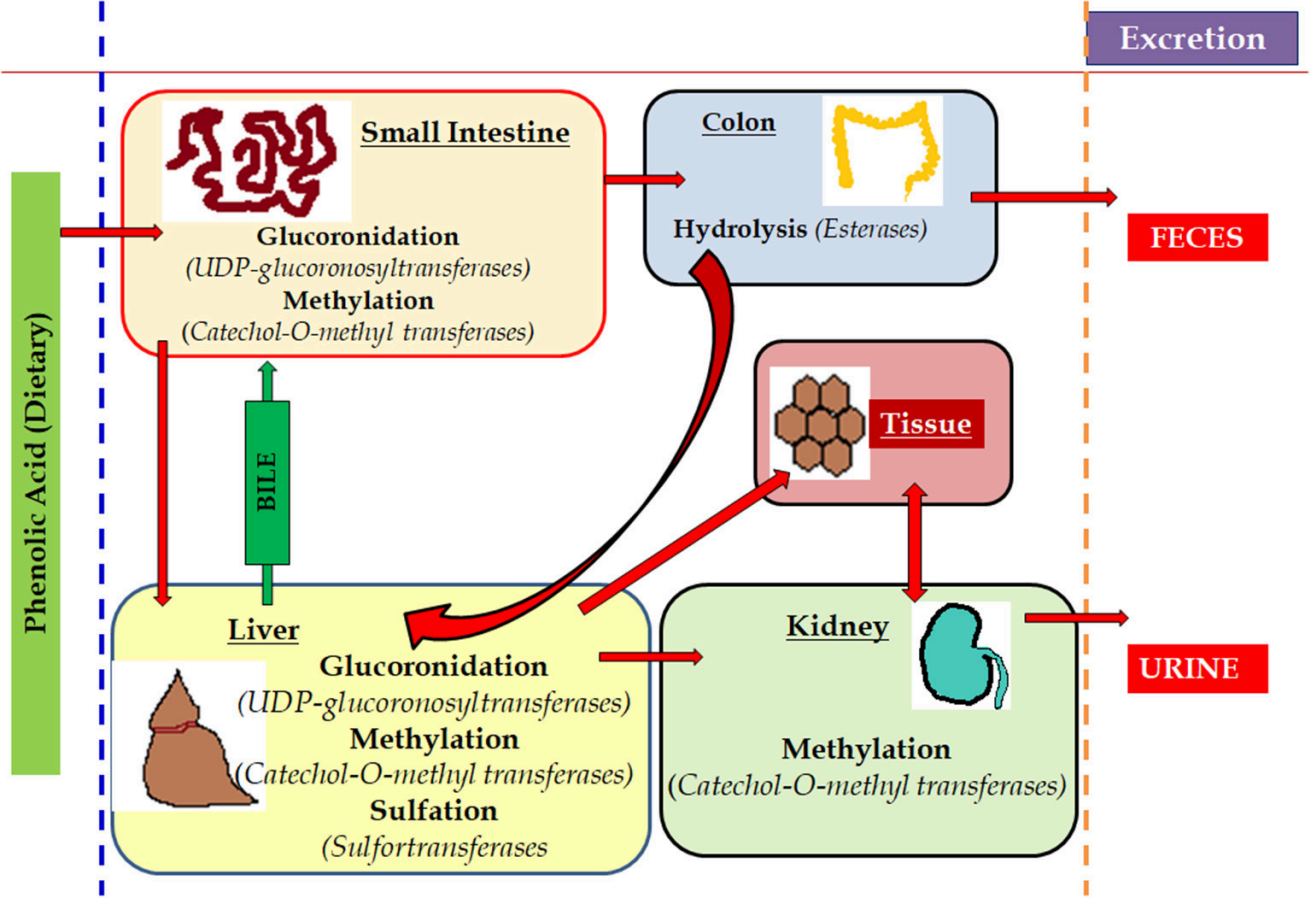
Metabolism of phenols in the living system. The metabolism of the dietary components rich in phenols is easily absorbed by various part of the animal body where the small intestine process and deviates the potent part to hepatic cells and remains are hydrolysed in colon and excreted via feces. Simultaneously, a part of it is methylated in kidney through liver and the last remains are excreted through urination. The red arrows mark is used to show the various route of metabolism of phenols.

To exert any pharmacological or biological actions, phenolic compounds are initially absorbed in the gastrointestinal tract (GIT) and thus make it bioavailable to circulating system. In the case of inadequate or no absorption through the GIT, they undergo biotransformation in the colon with the help of resident microbiota culture (Figure 7) (Filannino et al., 2015; Heleno et al., 2015; Gómez-Juaristi et al., 2018). Phenolic compounds offer health benefits including treating cancer, oxidative damage and inflammation. Literature supports their effectiveness against chronic pathogenic conditions like neurodegenerative and cardiovascular diseases (Heleno et al., 2015; Rangel-Huerta et al., 2015; Domínguez-Avila et al., 2017).

## Detailed Mechanism of Hepatoprotection

When the liver is exposed to alcohol, drugs, and pollutants, its progression toward damage initiates hepato steatosis, fibrosis, and cirrhosis. This exposure results in the death of hepatocytes and, as a consequence, the level of various liver enzymes and metabolites are altered, indicating the anomaly (Sheriff et al., 2017; Balderramo et al., 2018; Hu et al., 2018). Hepatocytes may be injured in various circumstances such as a toxic environment, alcohol, virus, fatty acid metabolism, or chronic antibiotics exposure. Transaminases and glutathione are reported to be prime candidates' marker in the line-up metabolism of bile when hepatocytes are damaged. The clinical condition of the hepatic environment can further be measured by levels of alkaline phosphatase (a key hepatic enzyme) in the serum (Culver, 2016).

Under these surroundings, scarring tissues try to replace the damages, and thus compromise vital liver functions like drug detoxification, secretion of the protein, albumin production, etc. (Anand and Garg, 2015; Baker, 2015). Metabolism, detoxification, and clearing of many drugs are blocked by the impaired liver (Bhattacharyya et al., 2014; Sheriff et al., 2017). Although there are several cited important bioactivity of phenolic compounds, the current discussion will primarily circle around the exploration of detailed mechanisms of actions, and further contributions of phenolics against various liver damages.

### Oxidative Stress and Hepatotoxicity

**Figure 8:**
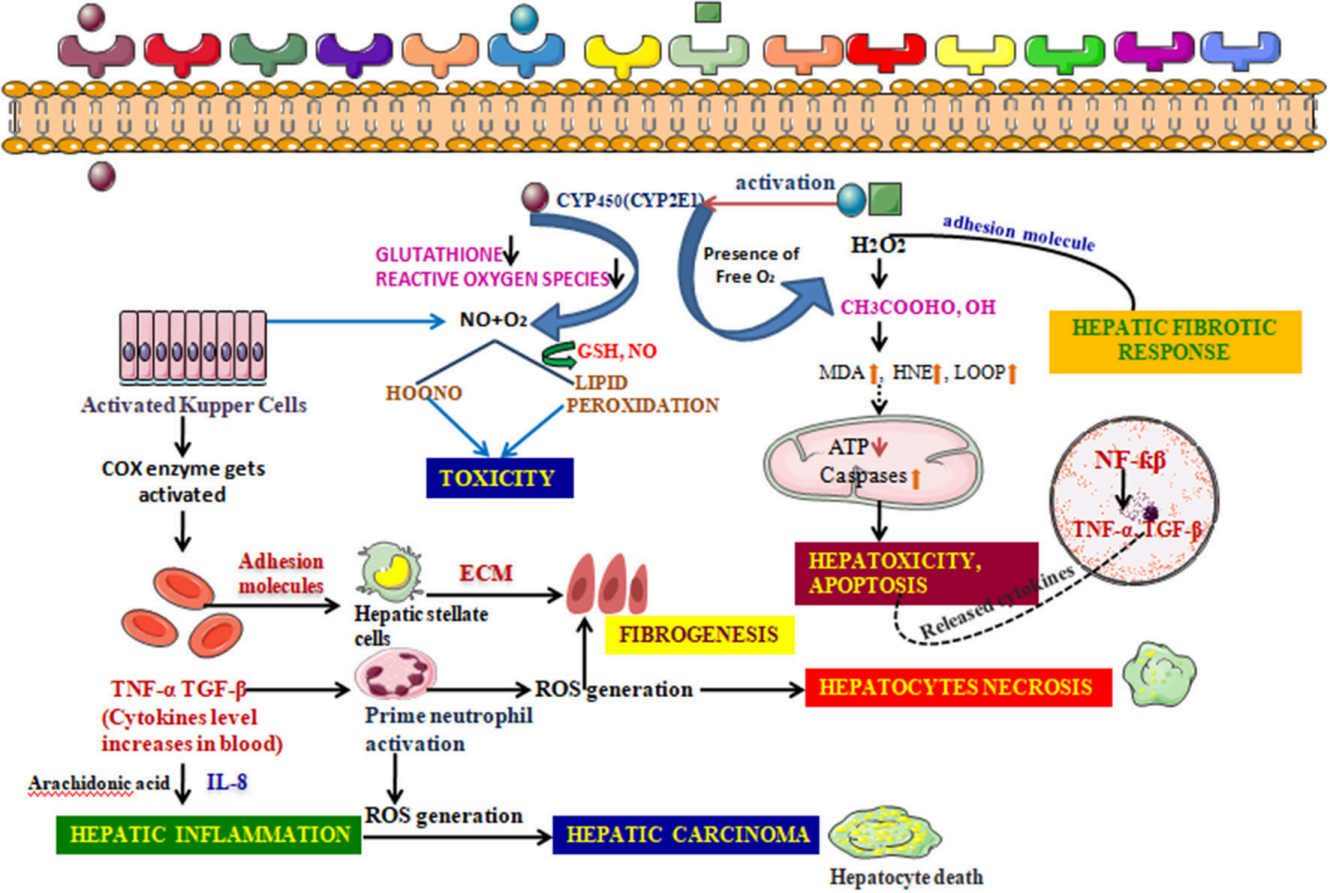
Detailed Mechanism of generation of hepatotoxicity. When the elicitors like alcohol, CCl_4_, enter the cell membrane they instigate various metabolic reactions activating the CYP systems viz; activating the endogenous glutathione enzyme, hydrogen peroxide. Formation of the reactive oxygen species are responsible for the lipid peroxidation reaction. A conjugation reaction takes place alongside this resulting in the deterioration in the ATP levels and elevation in the caspases levels. This clinical manifestation leads to the building up of hepatotoxicity and induces apoptosis. The nucleus also takes part in such build up by upregulating various transcription factors associated with inflammation. The adhesion molecules present in the cell membrane further create a hepatic fibrotic response by coupling with various reactive oxygen species. The activated Kupffer cells, on the other hand, further activate the prostaglandin by COX-2 and thus increases the cytokines level in the blood. These reactions are catalyzed by arachidonic acid. Such atrocities give rise to associated diseases with inflammation and further fibrogenesis. Hepatic necrosis is another condition imparted by the activated neutrophil which, though inactive during a normal state, increase in number when the cytokine level increases in the blood. The fatal condition, hepatic cirrhosis, is also encountered from hepatotoxicity, which is the additive effect of the prolonged inflammation and interaction with the ROS generation.

The liver, being a keen partner and prime neighbor of the GIT, is usually exposed to toxicity arising from a broad range of drugs, xenobiotics, and the stress mediated by reactive radicals formed during uncontrollable oxidation processes. Being a frequent target of such complex substances, and possessing a unique metabolism system, it hampers itself in the process of breaking them into simpler ones (Cederbaum, 2017). For instance, the large amount of bile acid produced during the oxidation of ethanol produces hepatocellular apoptosis by exciting Fas, an apoptotic element, expressing it from in the plasma membrane, which triggers apoptosis, resulting in cholestatic disease (Cederbaum, 2017). The liver also efficiently expresses main cytochrome P_450_ isoforms in response to various xenobiotics. CYP2E1 is one such that generates a reactive oxygen family, activates toxicologically central intermediates, and may be the critical alleyway by which these toxic chemical groups cause oxidative stress. Furthermore, kupffer cell, a specialized cell in the liver is activated in this process of metabolism. Both Kupffer cell activation and infiltration of neutrophil release reactive oxygen species (ROS), a range of inflammatory chemokines increasing the fold of hepatotoxicity (Figure 8) (Wang, 2015; Ahadpour et al., 2016).

*In vivo* and *in vitro* studies have demonstrated the promising preventative and therapeutic effects of plant phenolics in a range of liver diseases. Translational studies are extremely vital and indispensable for the application of phenolics in humans with liver diseases. Although literature in the PubMed database about clinical trials of phenolics in liver diseases are limited, encouraging beneficial effects of these phenols have been demonstrated, particularly in Non-Alcoholic Fatty Liver Disease (NAFLD). When working with the high fed diet, the AKT signal molecule responsible for fat metabolism is mutated in the model systems, placebo-inhibited trial of a purified form of anthocyanin in NAFLD patients, treating with the fixed amount of purified anthocyanin for 3 months significantly improved insulin resistance, in liver injury (Zhang et al., 2015a), and clinical evolution in such patients (Bischoff et al., 2018). In another double-blind clinical trial, dihydromyricetin, the main active ingredient of Ampelopsis grossedentata, improved glucose and lipid metabolism and showed anti-inflammatory effects in NAFLD (Chen et al., 2015b; Hou et al., 2015). When working with the hepatotoxic model system, the mice cohort which was treated with thioamino acetic acid showed significant recovery in its MAPK and AMPK level, the two important pathways, which impart cAMP and are a source of energy to the hepatocytes. This recovery was witnessed when a most studied flavonoid, curcumin, was administered at a dose of 118 μg/kg b.wt.

### Alcohol and Hepatotoxicity

**Figure 9:**
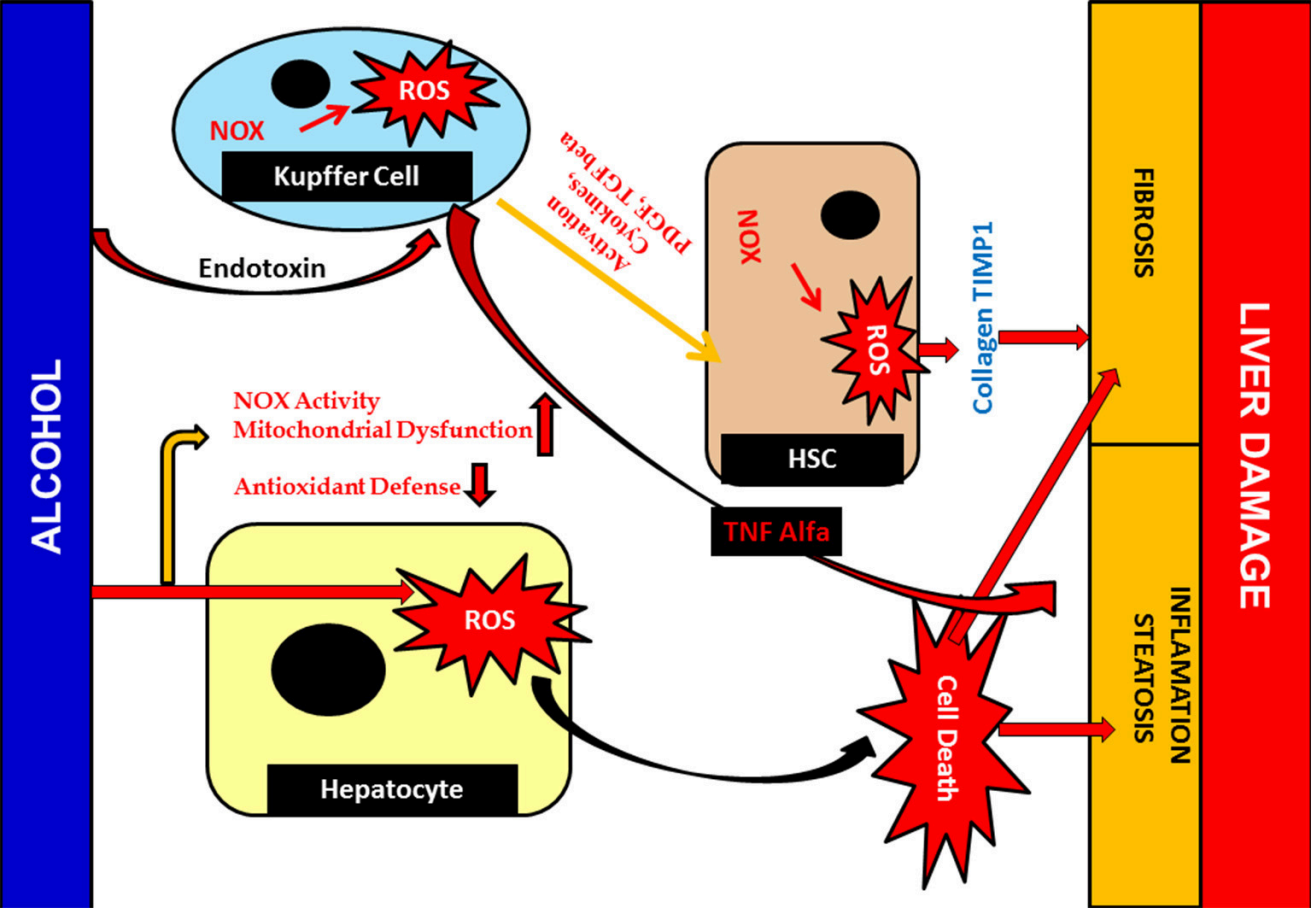
Alcohol mediated Hepatotoxicity. Hepatotoxicity caused by increased production of ROS; due to alcohol damages antioxidant defenses and mitochondrial function as well as structure. It leads to liver inflammation, fibrosis and steatosis. Cellular responses, which are sturdily involved in Kupffer cell may also activated due to action of ROS which contribute to an increase of inflammatory responses, resulting liver injury. Furthermore, activated Kupffer cells release ROS and cytokines that are crucial for HSC activation and inducing the pro-fibrogenic pathway.

Alcohol hinders the functional aspects of various tissue components and hepatocytes in particular. Alcohol diffuses, crossing the membrane barrier, and is distributed throughout the cell and tissue system, interacting with the major proteins and cellular component present in it (Li et al., 2016). Development of toxic molecules like reactive oxygen species (ROS) is another negative upshot of alcohol. In addition to ROS, it also produces acetaldehyde and nitric oxides, an extremely reactive and toxic by-product that chip into tissue damage (Madrigal-Santillán et al., 2015; Marshall, 2016). Nitric oxide (NO) is recognized as managing mitochondrial respiration and biogenesis amongst organelle. Under conditions of alcohol-mediated hepatic complications, mitochondrial respiration was hindered and, in turn, hypoxia occured. Simultaneously, nuclear factor-kappa β (NF-κβ), a transcription factor activation, takes place, in which it binds to iNOS promoter, an important NO, and aggravates the expression of iNOS (Figure 9) (Iwakiri and Kim, 2015; Starkel et al., 2016). Together, this entire environment amplifies the expression of inducible nitric oxide synthase (iNOS). iNOS joins hands in inducing hepatic fibrosis and the expression of inflammatory cytokines (Tacke and Zimmermann, 2014; Cassini-Vieira et al., 2015). iNOS increases two other factors in this process. Hypoxia-inducible factor-1 and its gene expression aid various connected hepatic anomalies viz; inhibition of mitochondrial respiration, impairment of mitochondrial fatty acid β-oxidation, and mitochondrial DNA damage (Chang et al., 2015; Suraweera et al., 2015).

Phenolics possess immense potentials in regulating the inflammatory cytokines, which are expressed in clinical conditions such as alcoholic liver diseases (Wan and Jiang, 2018; Xu et al., 2018). Puerarin, a known isoflavone, can excite the AMP-activated protein kinase (AMPK) phosphorylation in H4IIE cell lines suppressing the (m TOR) target proteins and 4E-binding protein (Zhao et al., 2016). This strategy aids in ameliorating the alcohol-based hepatotoxicity. Puerarin can also alleviate the hepatic necrosis due to its role in the AMPK pathway activation, scavenging activity, and lipid peroxidation inhibition (Wang et al., 2018a).

### Hepatotoxicity and Non-alcoholic Liver Disease

A majority of the metabolic disorders and their physiology related to hepatotoxicity have been studied over the years. Where sharp and clear possible elements that are responsible for chemical-induced toxicity, enzymes and protein-induced complications are considered, yet a fair amount of diseases related to metabolism remain unidentified. Such prognostic parameters include blood pressure, abdominal obesity, or potentially hyperglycaemia. They are collectively termed as the non-alcoholic fatty liver diseases (NAFLD) (Chalasani et al., 2018). This clinical situation is one of the most familiar and dormant forms of liver diseases, which accounts for the preliminary stage, but when left untreated this results in inflammation and, subsequently, can even lead to serious fibrosis and hepatocellular carcinoma (HCC), with high rates of mortality (Chen et al., 2015a).

Until now, the main drugs for the treatment of NAFLD in clinics are lipid regulating agents such as statins, which are not only toxic, but also aggravate the deposition of lipids in the liver, leading to serious liver injury (Arguello et al., 2015). Phenolics such as baicalin, epicatechin, and apigenin (Figure 5) have been reported to protect the liver from NAFLD, which are associated with their effects on insulin resistance and for signaling the way to anti-inflammation as well as antioxidant action (Sen and Chakraborty, 2017; Wan and Jiang, 2018).

Phenolic compounds can significantly regulate these NAFLD conditions. Apigenin, a flavone, is a well-studied phenolic compound that can check the lipid accumulation and oxidative stress induced by high-fat diet. It can abridge the inflammatory mediators but can simultaneously amplify various endogenous antioxidative enzymes actions like superoxide dismutase and glutathione peroxidase in the liver (Feng et al., 2017; Vergani et al., 2017). Dihydromyricetin, another important phenolic, exhibits its therapeutic effect on the improvement of glucose and lipid metabolism in patients with NAFLD, by blocking the phosphatidyl inositol 3-kinase, NF-κβ signaling pathway (Chen et al., 2015b).

### Hepatotoxicity and Inflammation

Liver inflammation is a state of the reaction in which the liver tissues send a constant stimulus, whether acute or chronic, in response to extrinsic and intrinsic factors hampering the liver status. Acute inflammation is a localized affair, where the liver tries to regain its previous configuration. It is the first line of defense, but when the liver cannot check these associated level of lymphocytes, vascular proliferation and tissue destruction become chronic and ultimately lead to fibrotic condition (Pawlak et al., 2015; Leyva-López et al., 2016).

**Figure 10:**
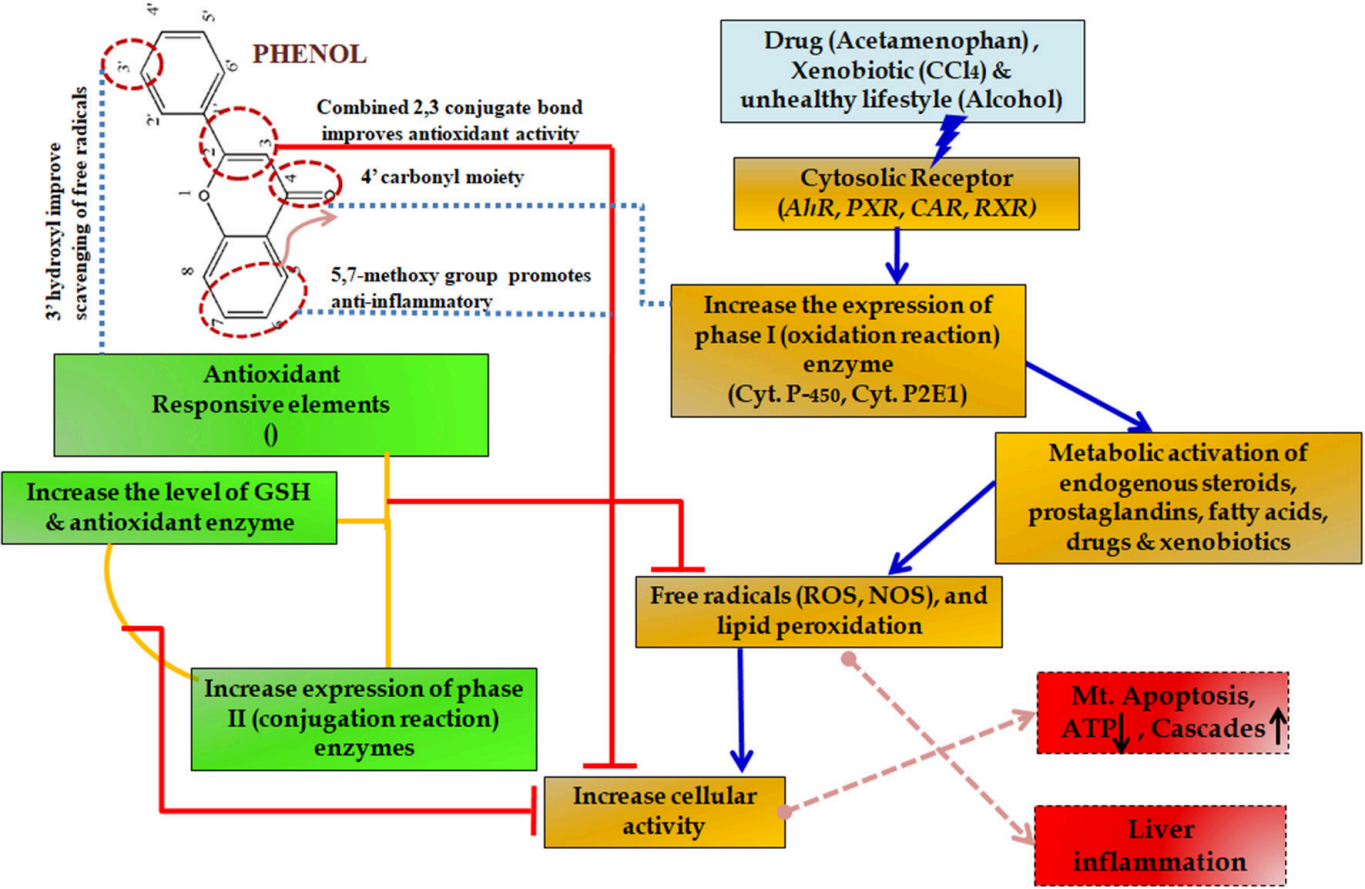
Protective effect of phenols in various metabolic pathways in liver diseases. The upward arrow indicating upregulation and down arrow indicating downregulation of the enzymes.

During such chronic conditions, specialized cells such as macrophages recruit more of the inflammatory mediators including interleukins and tumor necrosis factor (TNF)-α (Seki and Schwabe, 2015; Williams et al., 2016). This amplification altogether results in such a complex state that it leads to many degenerative diseases including severe cirrhosis and hepatic carcinoma (Czaja, 2014). For this reason, slowing down the inflammation process becomes essential. Initially, non-steroidal anti-inflammatory drugs (NSAIDs) are prescribed but the associated side effects include mild gastritis, renal failure and at times allergy due to hypersensitivity (Figure 10) (Pawlak et al., 2015).

Recent information on hepatic inflammation demonstrated the role of phenolics in protecting such inflammation. Phenolic compounds like hesperidin can act against inflammation by downregulating liver enzyme biomarkers such as aspartate amino transferase (AST) and alanine aminotransferase (ALT) primarily. It can also hold back oxidative stress and activation of T cells, which is a prime instigator of inflammation (Li et al., 2014). Hesperidin, a common Citrus flavonoid, further aids in the management of various proinflammatory recruiters viz; NF-κβ and α smooth muscle actin (α-SMA). Another well-known flavone, silymarin, is also a subclass of the family of phenolic compounds that works in patients with chronic hepatic carcinoma (González-Gallego et al., 2014).

## Regulation of Gene Expression by Phenolics

The chemical nature, physical properties, and dose ratio of a particular drug, along with an individual's gene expression profile, antioxidant status, and the capacity for regeneration are also crucial for cell injury. Several mechanisms are involved in the initiation of liver cell damage and aggravate ongoing injury processes (Guan et al., 2014; Ju and Tacke, 2016). Dysfunction of these vital cell organelles results in the impairment of dynamic equilibrium in homeostatic condition, thus resulting in intracellular oxidative stress with excessive formation of reactive oxygen species (Cannistrà et al., 2016; Ramachandran et al., 2018). Major causes of the hepatotoxic reactions by drugs are elevated ROS generation, oxidative stress and suppressed immune responses. Hepatotoxicity remains a major cause of drug withdrawal from the market. Recent examples in the USA and Europe are ximelagatran, nefazodone, nimesulide, ebrotidine, trovafloxacin, troglitazone, bromfenac, and so forth.

**Figure 11:**
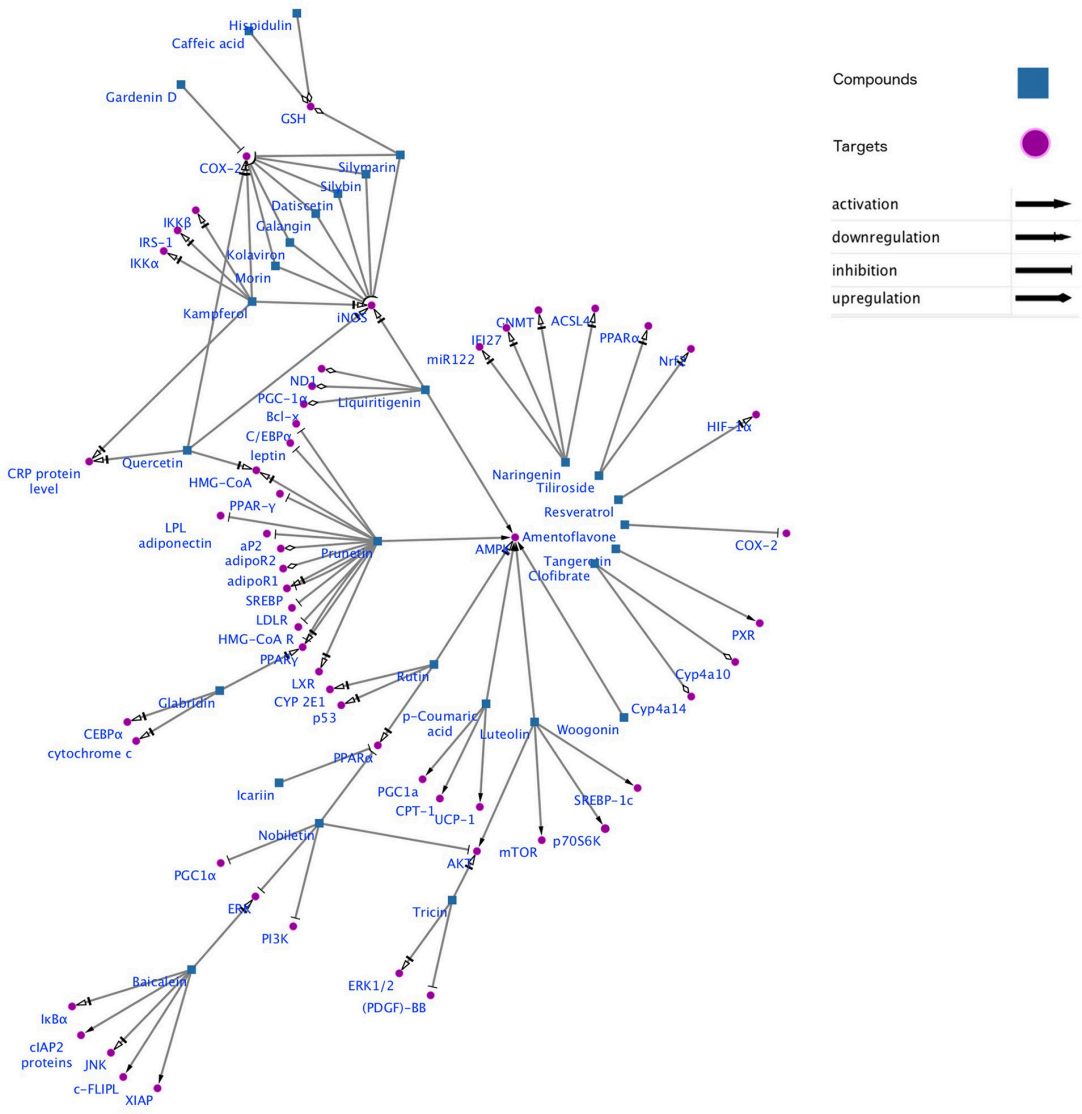
Gene networking showing hepatotoxicity mediated gene expression and subsequent mode of action of various natural products. This network was generated by a software Cytoscape version 3.6.1.

**Figure 12:**
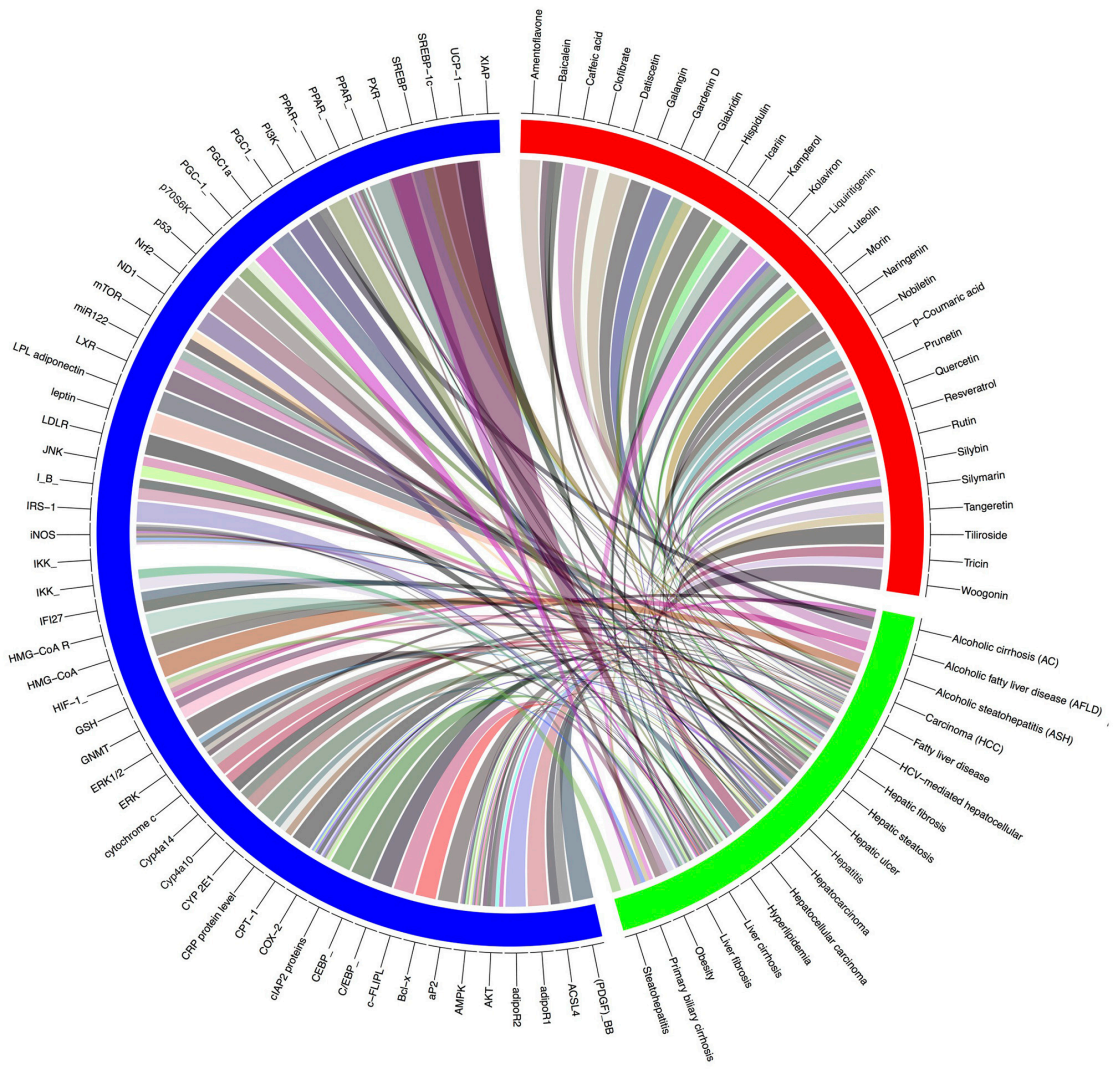
Gene-modeling showing various hepatic diseases and associated genes with it. A tool named Circus on shiny Circos server generated this image. The blue band is showing various genes responsible for pathophysiological conditions, the green showing various hepatic complications and the red band shows the bioactive natural compounds possessive hepatoprotective activity. Various shades indicating the degree of relatedness between the various bands.

Gene-metabolic networks are an advanced mode to construct a network with genes and metabolites specifically deregulated in different liver disease phenotypes. It compactly gives an overview of genes of interest, representative gene subsets that were involved in regulated signaling pathways, including tumor necrosis factor (TNF), P53, NF-κB, chemokine, peroxisome proliferator activated receptor (PPAR) and Toll-like receptor (TLR) signaling pathways associated with the physiology of various hepatic disease. Detailed information for the clinical status and associated genes in the hepatotoxicity are summarized in gene networking model Figures 11, 12. Gene regulation of a few bioactive phytocompounds is discussed below in Tables 1, 2.

**Table 1:** List of a few potent natural phenolics and their mode of action imparting hepatoprotective activity.

**Sl No**.	**Compound name**	**Sub category**	**Type of liver disease**	**Structure**	**Mode of action**	**References**
1	Apigenin	Flavone	Hepatic ischemia/reperfusion	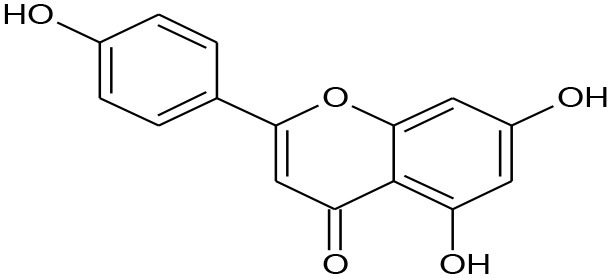	Up regulating BCL-2 levels	(Tsaroucha et al., 2016)
2.	Caffeic acid	Phenolic acids	Diabetic Liver injury	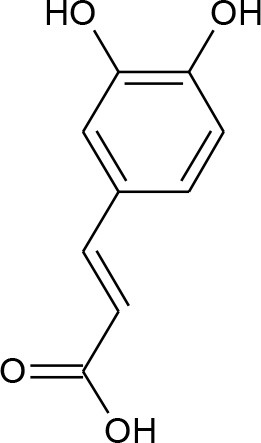	Lipid peroxidation and antioxidant enzymes	(Yilmaz et al., 2004)
3.	Catechin	Flavonols	Hepatic tissue injury	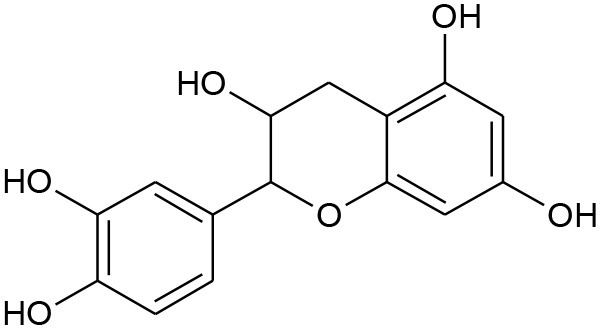	Antifibrotic and antioxidative	(Kobayashi et al., 2010)
4.	Curcumin	Curcuminoids	Non-alcoholic steatohepatitis	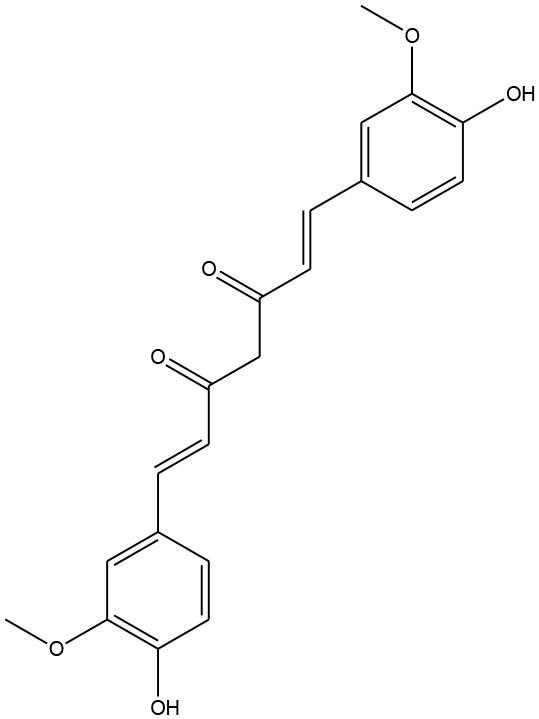	Immunomodulatory	(Nafisi et al., 2009)
5.	Epicatechin	Flavonoids	Diabetic liver injury	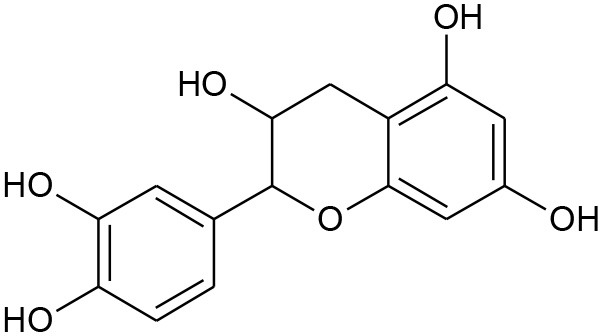	Lipid peroxidation and antioxidant enzymes Effects of (–)epicatechin, a flavonoid on lipid peroxidation and antioxidants streptozotocin-induced diabetic liver, kidney and heart.	(Terao et al., 1994)
6.	Ferulic acid	Phenolic acids	Carbon tetrachloride (CCl_4_)-induced acute liver injury	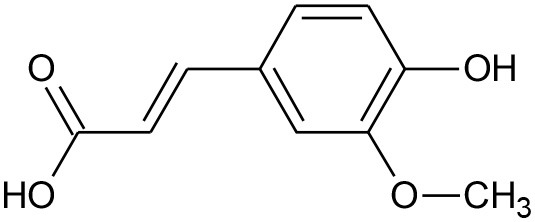	Antioxidant, anticancer, and anti-inflammatory	(Kim et al., 2011)
7.	Hyperoside	Flavonol	Liver injury	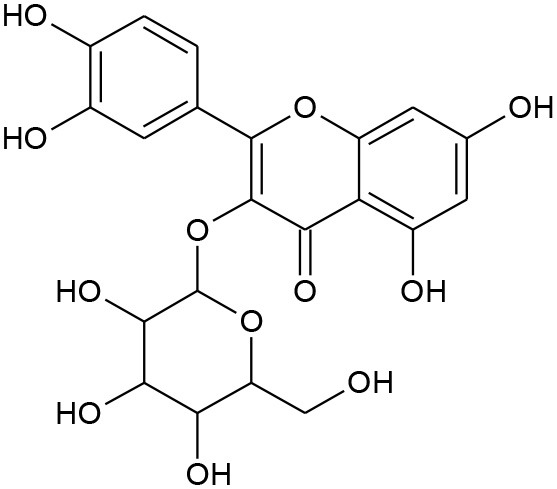	Enhancement of APAP clearance	(Choi et al., 2011)
8.	Icariin	Prenylated flavonol glycoside (Flavonoid)	Hepatic fibrosis	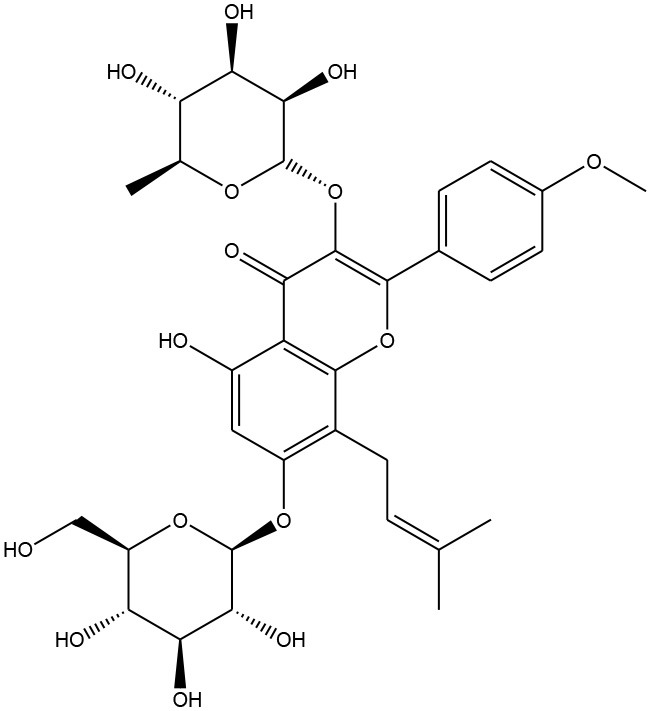	Anti-angiogenic and anti-autophagic	(Algandaby et al., 2017)
9.	Magnolol	Neo-lignan	Immune-related liver fibrosis	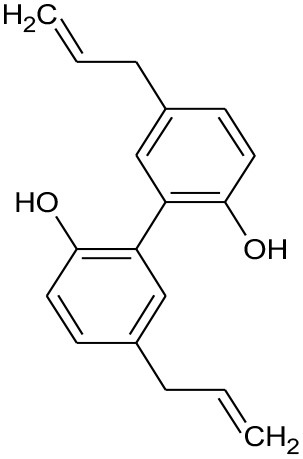	Anti-inflammatory and antioxidant effects	(Ogata et al., 1997; Lin et al., 2001)
10.	Morin	Flavonoid	Hepatic fibrosis	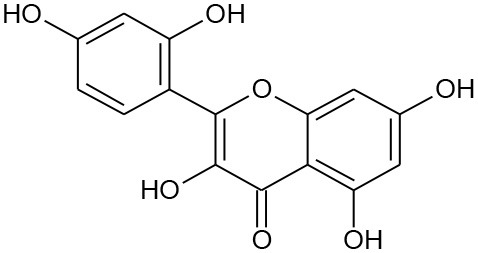	Suppressing canonical NF-κBsignaling.	(Sivaramakrishnan and Niranjali Devaraj, 2009; Madankumar et al., 2014)
11.	Naringenin	Flavanone	Hepatic inflammation	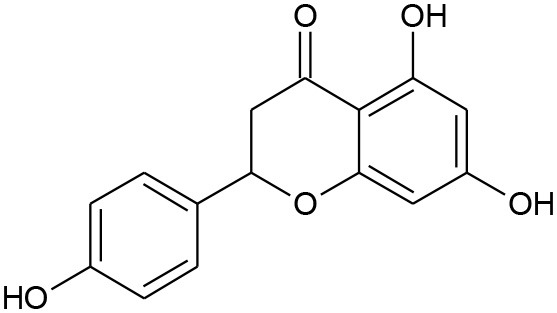	Activation of an Nrf2-mediated pathway	(Totta et al., 2004; Yen et al., 2009)
12.	Resveratrol	Stilbenoid	Alcoholic fatty liver	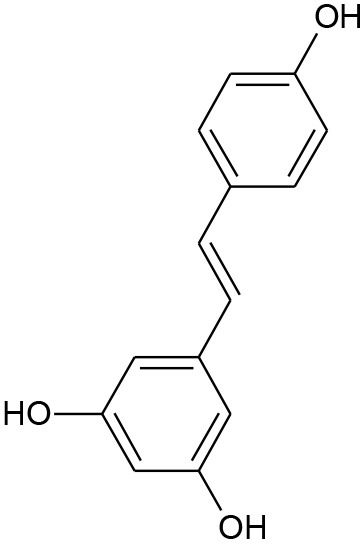	Inhibition of sirtuin 1 (SIRT1) and AMP-activated kinase (AMPK)	(Frémont, 2000; Baur and Sinclair, 2006)
13.	Wogonoside	Flavonoid	Hepatic fibrosis	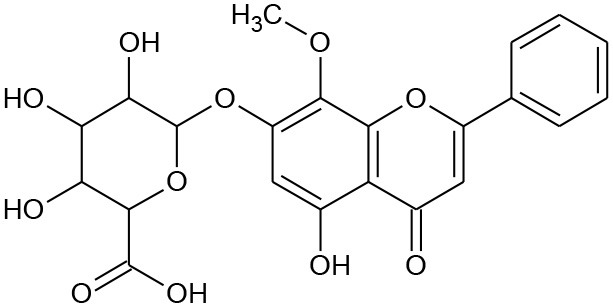	Antifibrotic	(Yang et al., 2013)

**Table 2:** Table showing various hepatic diseases and various genes and metabolites associated with it.

**Compound**	**Hepatic diseases**	**Clinical condition**	**Target**	**Family**	**Regulation**	**Involved in expression**	**References**
Amentoflavone	Microsomal lipid peroxidation	Fatty liver disease	COX-2	Inflammatory mediators	Inhibition	AP-1(downregulation)	(Arannilewa et al., 2006; Yadav et al., 2016)
Baicalein	Hepatic apoptosis, inflammatory liver injury	Acute liver apoptosis	IκBα, ERK and JNK	Inflammatory mediators	Downregulation	NF-κβ (down regulate)	(Liu et al., 2015; Meng et al., 2018)
Caffeic acid	Inflammatory liver injury	Acute liver failure	c-FLIPL, XIAP and cIAP2 proteins	Apoptotic protein	Activation	NF-κβ (down regulate)	(Shi et al., 2018)
	Inflammatory liver injury	Acute liver failure	c-FLIPL, XIAP and cIAP2 proteins	Apoptotic protein	Activation	TNF-α (down regulate)	(Wang et al., 2018b)
	Hepatic lipid peroxidation	Alcoholic fatty liver disease (AFLD)	Glutathione reductase(GSH)	Antioxidant enzymes	Increase	Microsomal ethanol-oxidizing system(increase)	(Chu et al., 2015)
Clofibrate	Hepatic excessive proliferation	HCV-mediated hepatocellular carcinoma (HCC)	Cyp4a10 and Cyp4a14	mRNA expression of factors	Increase	Acox1, Ech1, and Ehhadh (increased) Lipe and Pnpla2 (increased)	(Moody and Reddy, 1978; Bogdanska et al., 2018)
Galangin	Microsomal lipid peroxidation	Fatty liver disease	COX-2 and iNOS	Inflammatory mediators	0 inhibition	NF-κB (downregulation)	(Ren et al., 2016)
Gardenin D	Microsomal lipid peroxidation	Fatty liver disease	COX-2	inflammatory mediators	Inhibition	AP-1(downregulation	(Toppo et al., 2017)
Glabridin	Chronic inflammatory liver disease	Acute or chronic hepatitis and	PPARγ (peroxisome proliferator-activated receptor gamma)				(Thakur and Raj, 2017; Li et al., 2018)
	Chronic inflammatory liver disease	Hepatic steatosis	CCAAT enhancer binding protein alpha (CEBPα)	Inflammatory mediators	Downregulation	Phosphoenol pyruvate carboxykinase and glucose 6-phosphatase (downregulate)	(Namazi et al., 2017)
			Cytochrome c,				(Lin et al., 2017)
Hispidulin	Hepatic lipid peroxidation	Alcoholic fatty liver disease (AFLD)	Glutathione reductase(GSH)	Antioxidant enzymes	Increase	Microsomal ethanol-oxidizing system(increase)	(Wu and Xu, 2016);(Han et al., 2018)
Icariin	Hepatic excessive proliferation	HCV-mediated hepatocellular carcinoma (HCC)	PPARα	mRNA expression of factors	Inhibition	Cpt1a, Acat1, Acad1 and Hmgcs2 (increased)	(Lee et al., 1995);(Lu et al., 2014)
Kaempferol	Fatty liver diseases	Liver fibrosis	iNOS, COX-2 and CRP protein level	Inflammatory mediators	Downregulation	NF-κβ (down regulate)	(García-Mediavilla et al., 2007; Kashyap et al., 2017)
	Fatty liver diseases	Liver fibrosis	(IRS-1) (IKKα) and (IKKβ).	Inflammatory mediators	Downregulation	kappa-β (NF-κB), (TNF-α) and (IL-6) (down regulate)	(Dong et al., 2017)
Kolaviron	Liver inflammation	Primary biliary cirrhosis	COX-2 and iNOS	Inflammatory mediators	Inhibition	NF-κB and AP-1 (downregulation)	(Adaramoye and Lawal, 2015);(Awogbindin et al., 2015)
Liquiritigenin	Hepatic failure	Liver cirrhosis and hepatocellular carcinoma	PGC-1α, ND1, and Bcl-x	Metastasis mediators	Upregulation	Apoptosis (downregulate)	(Yu et al., 2015);(Li et al., 2018)
		Liver cirrhosis and hepatocellular carcinoma	AMPK		Activation	FXR (promote expression)	(Teng et al., 2016)
Luteolin	Liver injury	Fatty liver development	SREBP-1c	Transcriptional factors	Activation	Cholesterol biosynthesis (activation)	(Seydi et al., 2018)
		Hepatic steatosis	AMPK	Energy sensor	Activation	ATP-producing catabolic pathways	(Lee et al., 2006);(Cummins et al., 2018)
						, Such as FA oxidation(activation)	(Kwon and Choi, 2018)
		Liver cirrhosis and hepatocellular carcinoma	iNOS	Inflammatory mediators	Downregulation	NF-Kβ (down regulate)	(Jung et al., 2017)
	Hepatic diseases	Hepatic fibrosis	AKT, mTOR and p70S6K	Energy sensor	Activation	TGFβ1-simulated phosphorylation of AKT(downregulate0	(Domitrović et al., 2009; Panahi et al., 2018; Wan and Jiang, 2018)
Morin	Microsomal lipid peroxidation	Fatty liver disease	COX-2 and iNOS	Inflammatory mediators	Inhibition	NF-κB (downregulation)	(Fang et al., 2003);(Shankari et al., 2010)
Naringenin	HIV-1/HCV co-infective liver disease	HCV-mediated hepatocellular	ACSL4, GNMT, IFI27, and miR122		Downregulation	NF-Kβ (down regulate)	(Jain et al., 2011; Hernández-Aquino and Muriel, 2018)
		Carcinoma (HCC)				TNF-α (down regulate)	(Hernández-Aquino and Muriel, 2018)
Nobiletin	Chronic inflammatory liver disease	Liver cancer	PPARα and PGC1α		Inhibiting adhesion,		(He et al., 2016; Kim et al., 2017; Wu et al., 2018)
	Chronic inflammatory liver disease	Liver cancer	ERK and PI3K/Akt	Metastasis mediators	Invasion, and migration	iNOS and COX-2, TNF-α (down regulate)	(Yuk et al., 2018)
Prunetin	Lipid accumulation in the liver	Hyperlipidemia	AMPK	Metastasis mediators	activation	HMG-CoA R (inactivates)	(Wei et al., 2018)
	Adipogenesis in the liver	Fatty liver disease	PPARγ, C/EBPα, SREBP, aP2,	Adipogenic genes	Inhibition	LDLR (promote expression)	(Chen et al., 1998);(Ding et al., 2016)
			LPL adiponectin, and leptin				(Zhang et al., 2007)
	Adipogenesis in the liver	Fatty liver disease	SREBP, PPARγ, LXR, and HMG-CoA	Lipid Metabolism-related genes	Suppressed	LDLR (promote expression)	(Walle, 2007)
	Adipogenesis in the liver	Fatty liver disease	Adipor1, adipoR2	Adiponectin receptors	Induction	AMPK induction	(Wei et al., 2018)
Quercetin			iNOS, COX-2 and CRP protein level	Inflammatory mediators	Downregulation	NF-Kβ (down regulate)	(Gupta et al., 2010; Kumar et al., 2016)
Rutin	Hepatic diseases	Hepatocarcinoma	PPARα, AMPK activity,	Metastasis mediators	Downregulation	SREBP-1(down regulate)	(Pan et al., 2014)
		Fatty liver disease					
		Obesity					
		Hyperlipidemia					
	Hepatic diseases	Hepatocarcinoma	p53 and CYP 2E1	Reactive metabolic	Downregulation	ROS (down regulate)	(Mansour et al., 2017);(Nazeri et al., 2017)
		Fatty liver disease		Trichloromethyl radicals			
		Liver cirrhosis		Trichloromethyl radicals			
Silibin	Microsomal lipid peroxidation	Fatty liver disease	COX-2 and iNOS	Inflammatory mediators	Inhibition	NF-κB (downregulation)	(Yu and Ren, 2008; Hernandez-Rodas et al., 2015; Younossi, 2017)
Silymarin	Hepatic centrilobular necrosis	Paracetamol toxicity	Glutathione reductase (GSH)	Antioxidant enzymes	Increase	Microsomal ethanol-oxidizing system(increase)	(Lieber et al., 2003);(Ni and Wang, 2016);(Abenavoli et al., 2018)
		Steatohepatitis, hepatic fibrosis					(Boari et al., 1981);(Pradhan and Girish, 2006);(Vargas-Mendoza et al., 2014)
	Steatosis	Chronic hepatitis C	COX-2 and iNOS	Inflammatory mediators	Inhibition	NF-κB and AP-1 (downregulation)	(Saller et al., 2001; Jose et al., 2011)
Tangeretin	Chronic inflammatory liver disease	Primary biliary cirrhosis	Pregnane X Receptor(PXR)	Nuclear receptor gene	Activation	NF-κβ (down regulate)	(Di Carlo et al., 1999; Omar et al., 2016)
		Liver fibrosis				TNF-α (down regulate)	(Fracanzani et al., 2008)
Tricin	Liver inflammation	Liver cirrhosis	ERK1/2 and Akt	Downstream signaling molecules	Supress	Blocking cell cycle progression	(Seki et al., 2012; Arulselvan et al., 2016)
	Hepatic diseases	Hepatocellular carcinoma	(PDGF)-BB	Platelet-derived growth factor	Inhibition	Blocking cell cycle progression	(Malvicini et al., 2018)

### Apiginin

Apigenin, a plant flavone, can improve hepatic health during severe liver disease conditions by down-regulating Nrf2-signaling and up-regulatingBCL-2 apoptotic pathway (Tsaroucha et al., 2016).

### Caffeic Acid

It is chemically 3,4-dihydroxycinnamic acid that occurs in a diet of fruits, green tea, wine, and coffee bean components. Caffeic acid showed potential antioxidant and anti-inflammatory properties and is effective in treating major liver hitches (Kim et al., 2018). It can modulate the expression of kelch-like ECH-associated protein-1 (Keap1), a hepatic carcinoma factor, by interacting with Nrf2 binding site and restraining it from binding to Keap1 and elevating the expressions of vital antioxidative signals like HO-1 (Yang et al., 2017).

### Catechin

Catechin from green tea extracts, selective seeds, and fruits. It is categorized by the presence of a hydroxyl moiety at C3, C5, and C7 position of at A ring, and again in C3 and C4 of the B ring. Catechin with anti-hyperlipidemic property helps in treating diverse clinical conditions associated with non-alcoholic fatty liver diseases where abnormalities in protein and lipid metabolism play the prime role in pathophysiology of the liver (Sun et al., 2015; Pezeshki et al., 2016).

### Curcumin

It exerts its protective and therapeutic effects in oxidative coupled liver diseases by suppressing proinflammatory cytokines, lipid peroxidation products, hepatic stellate cells, and Akt activation. Curcumin ameliorates oxidative stress induced expression of Nrf2, SOD, CAT, and GSH. Curcumin acts as a free-radical scavenger over the activity of different kinds of ROS via its active phenolic pharmacophore, β-diketone and methoxy group (Nabavi et al., 2014).

### Epicatechin

It is a flavan-3-ol found in edible plant products like cocoa and other varieties of plant foods. Epicatechin plays an important role in lipid metabolism in fatty liver condition and hypercholesterimia (Cordero-Herrera et al., 2015). It can down-regulate important liver enzymes like SGPT and SGOT, which increases its liver anomalies (Shanmugam et al., 2017).

### Ferulic Acid

It is the most abundant phenolic acid in plants that has potent antioxidant ability to freeze the activity of the free radicals like NO, ${\text{O}}_{2}^{-}$. It exhibits prevailing anticholestatic action against liver cholestasis by inhibiting extracellular matrix related gene expression and also by disruption of the Smad signaling pathways and extracellular signal-regulated kinases (Gerin et al., 2016). It sometimes activate the AMPK or the MAPK signaling pathway by enhancing lipid metabolism (Cheng et al., 2018). Several reports also confirmed the mode of action of ferulic acid is mediated by regulating the expression of several physiological factors viz; PPAR-α, CPT-1α toward lipid oxidation and this action is very important in treating fatty liver diseases (Kim et al., 2011).

### Hyperoside

It is a significant flavonoid that can fuel up the expression of diverse endogenous antioxidant enzymes and can quench free radicals formed during the metabolism of xenobiotics in the liver. Further, the capacity of hyperoside to regulate detoxifying enzymes phase II makes it potent as these enzymes are the prerequisite for liver during the initial round of oxidation. It helps in mitigating liver fibrosis by activating the Nrf2 signaling pathway, meant for neutralizing oxidants, when studied in CCl_4−_induced hepatotoxicity (Wang et al., 2016; Xie et al., 2016; Zou et al., 2017).

### Iccarin

It is reported from genus *Epimedium* and has been shown to delay the fibronectin and collagen accumulation in renal interstitial tissues and mesengial cells of rat model (Algandaby et al., 2017). Several published reports confirmed its protective role in inflammation blocking TNF-α and IFN-γ signaling pathway (Sinha et al., 2016). Other important protective actions of iccarin comprises of modulating expression of toll-like receptor and inhibition of the mitogen activated protein kinase (MAPK) (Mochizuki et al., 2002).

### Magnolol

Magnolol from *Magnolia officinalis* is an important phenolic compound that maintains the oxidative balance during hepatotoxicity in galactosamine-injured mice models. Magnolin, another phenolics from same plant was reported to have ameliorating activity in lipid build up, insulin resistance and also in hepatic inflammation, when hepatocytes are exposed to free fatty acid *in vitro* (Tian et al., 2018).

### Morin

Morin, is a naturally occurring 2′,3,4′5,7-penta-hydroxyflavone, present in mulberry, tartary buckwheat, jackfruit, green tea, orange, and in many dietary plants. It exerts beneficial effects on metabolism by suppressing canonical NF-Kβ signaling (Caselli et al., 2016; Sinha et al., 2016).

### Naringenin

Naringenin, a natural flavonoid, possesses antioxidant, anticancer and anti-inflammatory activity (Chtourou et al., 2015). Naringenin exhibits very little antioxidant action directly as a scavenger, yet it helps in upregulating of Nrf2 pathways and thus upholds the normal redox of the cell even in clinical conditions where prooxidants and reactive oxygens are formed as a of damage mechanism in hepatocytes, (Esmaeili and Alilou, 2014).

### Resveratol

Resveratrol, a 3,5,4′-trihydroxystilbene polyphenolic compound, is available in edible plants and selected fruits like grapes. It can control a specialized mammalian homolog, sirtuins (SIRT) (Andrade et al., 2014). Over expression of this homolog helps in treating non-alcoholic related fatty liver disease by regulation lipogenesis. Resveratrol is associated with considerable reduction in various liver enzymes, cytokines, and also transcriptional factors like nuclear factor κB. It alleviates the nuclear factor-κB (NF-κB) expression following the stimulation of its inhibitor IκBα (Zhang et al., 2015b).

### Wogoloside

It is another flavone that imparts hepatoprotective activity via different facilitating lipid metabolism by increasing oxidation process. AMPK signaling to bestow its effectiveness by various modules (Wang et al., 2015).

## Computational Study for Bioactive Phenolic Compounds

*In silico* appraisal presently happens to be a pronounced method of evaluation in various biological research these days. It has the benefit of low cost, fast execution, and the most constructive face of such study is to diminish the animal usage in various toxicity screening. PASS prediction assay (Lagunin et al., 2000), which is highly studied these days, is based on primarily structure-activity relationships investigation of the training set, that generally contains more than 200,000 compounds showing at least 3,700 type of biological actions that interestingly allows to estimate if a phytochemical compound has a particular effect (Dei et al., 2013). Lipinski's Rule of Five (Lipinski, 2004) is another method that can be applied to all the phenolic compounds to evaluate their drug likeness and pharmacological properties. Such information is very helpful in accessing the phenolic compounds as potential drug leads that can act as natural therapeutics. Only the compounds satisfying the Lipinski's criteria are further considered for additional computational operations. Compounds that cleared the Lipinski's barrier were prepared for docking studies by their energy minimization in Marvin Sketch. Receptor-ligand interaction study using the Hex docking tool (Macindoe et al., 2010) are also another mode of interaction study. Various amino acids of the target protein interaction with the lead compound are studied with respect to their bond length and bond angle. Hence, the reported phenolic compounds can thus be studied as good prospective options for their use as medicine that targets various proteins for hepatic treatment. Reports of phosphorylated flavonoids i.e., iccartin is extensively studied for the potent target TGF-β, where the score of molecular docking was reported 0.28 which was more than the marketed standard ursidiol 0.23 (Wheng et al., 2016).

*Insilico* studies have its implication in various pharmacological studies. From the initial protein, study to gene expression analysis related to any diseases can be carried out by the concept of pharmacogenomics. Phenolic compounds as hepatoprotective have been reported in the work of Kaveri, 2017, with *insilico* approach. The work was carried out on a group of newly synthesized acetylated phenolics. A good number of target proteins of hepatic anomaly have been reported when target fishing was performed (Liu et al., 2017); which not only predicted the probable important target but directed the study of those prospective targets in understanding the mechanism of that disease. This mode thus supports the traditional uses for hepatic disorders and thus can suggest major bioactive phenolic compounds as contributors to produce ethnopharmacological effect.

## Future Prospects

Natural products and specially plant phenolics have become a promising therapeutic alternative and prospective replacement of conventional marketed drug in practice due to their effectiveness, minimal side effects, and protective properties. Furthermore, their dietary nature and availability is a bonus, and gives all the more reason to decline those generally available drugs that also cause toxicity to cells. Remarkable phenolics like curcumin and resveratrol are pharmacologically tested chemoprotective agents against treatment of hepatic carcinoma. Though widely held natural products evaluated until now are generally non-toxic in nature, a few studies on toxicity regarding certain natural products are also highlighted these days. As a result, appropriate selection of the natural based drug is also obligatory. All the important phenolics with their derivatives, though studied and well reported for, have not yet been fully analyzed for their immense therapeutic usage, as there are not enough studies available regarding them. Components of such compounds in the diet varies with temperature and cultivation process. Furthermore, variation in the physicochemical properties could result from different modes of production of such plants, including agricultural and environmental factors. Many pharmacological reports have demonstrated that phenols have a variety of therapeutic effects, including anti-cancer, anti-diabetic, anti-obesity, immunomodulatory, cardioprotective, hepatoprotective, and neuroprotective effects through antioxidant and anti-inflammatory activities. However, additional studies are required to understand biological functions and compositions of many phenols, such as iccartin and morin, in more detail. Understanding biological function, composition, and therapeutic effects could help prevent adverse effects from long-term administration of phenolic compounds, and develop health promoting properties. It is envisaged from this presented review that plant based phenolics will not only reduce the risk of hepatopathy, but will also endow a sure substitute that can be used for various hepatotoxicity mediated diseases.

## Author Contributions

PS prepared the initial draft and graphical representation for figures. AT finalized the manuscript and supervised as a whole. RN worked on the graphical representations and carried out various literature survey studies. JS worked on gene network modeling and the gene expression study. MC worked on bioinformatics and the phenolic study in hepatic disease. SS and LN provided significant input into the chemistry part of this review, editing, and finalizing the draft.

### Conflict of Interest Statement

The authors declare that the research was conducted in the absence of any commercial or financial relationships that could be construed as a potential conflict of interest.
